# Adverse Birth Outcomes Due to Exposure to Household Air Pollution from Unclean Cooking Fuel among Women of Reproductive Age in Nigeria

**DOI:** 10.3390/ijerph18020634

**Published:** 2021-01-13

**Authors:** Jamie Roberman, Theophilus I. Emeto, Oyelola A. Adegboye

**Affiliations:** 1 Public Health & Tropical Medicine, College of Public Health, Medical and Veterinary Sciences, James Cook University, Townsville, QLD 4811, Australia; jamie.roberman@my.jcu.edu.au (J.R.); theophilus.emeto@jcu.edu.au (T.I.E.); 2 Australian Institute of Tropical Health and Medicine, James Cook University, Townsville, QLD 4811, Australia

**Keywords:** cooking fuel, household air pollution, preterm births, perinatal mortality, low birth weight, stillbirth, Nigeria

## Abstract

Exposure to household air pollution (HAP) from cooking with unclean fuels and indoor smoking has become a significant contributor to global mortality and morbidity, especially in low- and middle-income countries such as Nigeria. Growing evidence suggests that exposure to HAP disproportionately affects mothers and children and can increase risks of adverse birth outcomes. We aimed to quantify the association between HAP and adverse birth outcomes of stillbirth, preterm births, and low birth weight while controlling for geographic variability. This study is based on a cross-sectional survey of 127,545 birth records from 41,821 individual women collected as part of the 2018 Nigeria Demographic and Health Survey (NDHS) covering 2013–2018. We developed Bayesian structured additive regression models based on Bayesian splines for adverse birth outcomes. Our model includes the mother’s level and household characteristics while correcting for spatial effects and multiple births per mother. Model parameters and inferences were based on a fully Bayesian approach via Markov Chain Monte Carlo (MCMC) simulations. We observe that unclean fuel is the primary source of cooking for 89.3% of the 41,821 surveyed women in the 2018 NDHS. Of all pregnancies, 14.9% resulted in at least one adverse birth outcome; 14.3% resulted in stillbirth, 7.3% resulted in an underweight birth, and 1% resulted in premature birth. We found that the risk of stillbirth is significantly higher for mothers using unclean cooking fuel. However, exposure to unclean fuel was not significantly associated with low birth weight and preterm birth. Mothers who attained at least primary education had reduced risk of stillbirth, while the risk of stillbirth increased with the increasing age of the mother. Mothers living in the Northern states had a significantly higher risk of adverse births outcomes in 2018. Our results show that decreasing national levels of adverse birth outcomes depends on working toward addressing the disparities between states.

## 1. Introduction

Adverse birth outcomes (ABO) including low birth weight (LBW), preterm birth, and stillbirths represent an unmet public health need in Nigeria. The World Health Organization (WHO) classes infant birth weight of less than 2.5 kg as LBW [1]. LBW is a key risk factor for morbidity and mortality as well as being a predictor of survival, normal growth, and cognitive development in children [2,3]. In 2015, Nigeria had the third-highest number of neonatal deaths, and an estimated 313,700 stillborn deaths, the second-highest globally, and the highest number of maternal mortalities [4]. Hence, there is a need to explore the high incidence rate of these ABO in Nigeria compared with other countries.

Household air pollution (HAP) is a prominent global public health concern and is a leading environmental health risk globally [5]. In 2016, HAP was responsible for 7.7% of the global mortality [6]. It is estimated that 3.8 million premature deaths occur each year from illnesses attributable to HAP [7]. Of these deaths, 27% were due to pneumonia, 27% to ischaemic heart disease, 20% to chronic obstructive pulmonary disease (COPD), 18% to stroke, and 8% to lung cancer [7]. HAP has also been linked to pulmonary tuberculosis [8], eclampsia [9], and cataracts [10]. Regional variations in the particulate composition of HAP has been reported within countries [11] and between countries [12], and may also depend on locally accessible fuel type within regions [12,13]. Within Nigeria, geographic factors also impact the rate of adverse birth outcomes as the North West region has a significantly higher risk for LBW [3]. Hence, it is important to highlight the effect of regional variation on the prevalence of ABO due to HAP. Additionally, some studies have shown that parental employment, education level, and other indicators of socio-economic status are associated with LBW [14,15].

A primary source of HAP is the use of unclean fuel in cooking [7]. Common chronic respiratory symptoms, such as congestion and cough, are directly associated with the hours spent cooking food [16]. Using unclean fuel is especially common in developing countries where biomass fuel sources are more affordable than healthier alternatives such as electricity, natural gas, and liquefied petroleum gas [17]. Biomass fuel includes wood, crop residue, charcoal, coal, and dung [7]. The use of this type of fuel is most prevalent in Africa and Southwest Asia where over 60% of households cook with unclean fuels [18,19], and there is evidence of geographical disparities in its use [20]. Nearly 70% of the Nigerian population cook with unclean fuel [21].

Global evidence suggests an association of HAP with increased pregnancy complications [22], preterm birth [23], and elevated risk of stillbirth [24,25]. It is hypothesized that pollutants, including carbon monoxide, contained in HAP can be inhaled and absorbed into the mother’s blood which could detrimentally affect the fetus [26,27]. HAP likely potentiates ABO by promoting a pro-hypoxic phenotype including increased expression of Hofbauer cells, syncytial knots, and a compromised chorionic vascular density in utero [28]. Uterine hypoxia has also been associated with morbidity and mortality [29]. Both Hofbauer cells [30] and syncytial knots [31,32] have been associated with ABOs such as pre-eclampsia and intrauterine growth retardation, while impaired chronic vascular density is linked to embryonic death [33]. In Nigeria, a limited randomized control trial demonstrated the increased risk of chronic hypoxia in pregnant women using unclean cooking fuel compared to those using clean cooking fuel [28]. Another randomized trial showed an intervention of ethanol as cooking fuel significantly increased mean birth weight and gestational age at delivery [34]. This may also mitigate the risk of cardiovascular disease such as hypertension by downregulating the expression of tumor necrosis factor alpha, interleukin 6, and interleukin 8 in pregnant women [35]. 

Here, we explore the impact of HAP on adverse birth outcomes, such as stillbirths, pregnancy duration, and LBW in Nigeria. We will evaluate the geographic distribution of the association to identify regions with more significant HAP effects on births. This study will add to the existing literature by analyzing the impact of HAP on ABO in Nigeria while accounting for geographic heterogeneity.

## 2. Materials and Methods 

### 2.1. Data Source, Setting, and Variables

This study was based on the Nigerian Demographic and Health Survey (NDHS) phase 6 2013–2018 (hereafter, NDHS2018). NDHS is a cross-sectional nationally representative survey that has been conducted every five years in Nigeria since 2003 [36]. The NDHS is funded by the United States Agency for International Development as part of the worldwide Demographic and Health Surveys Program [36]. 

**Figure 1:**
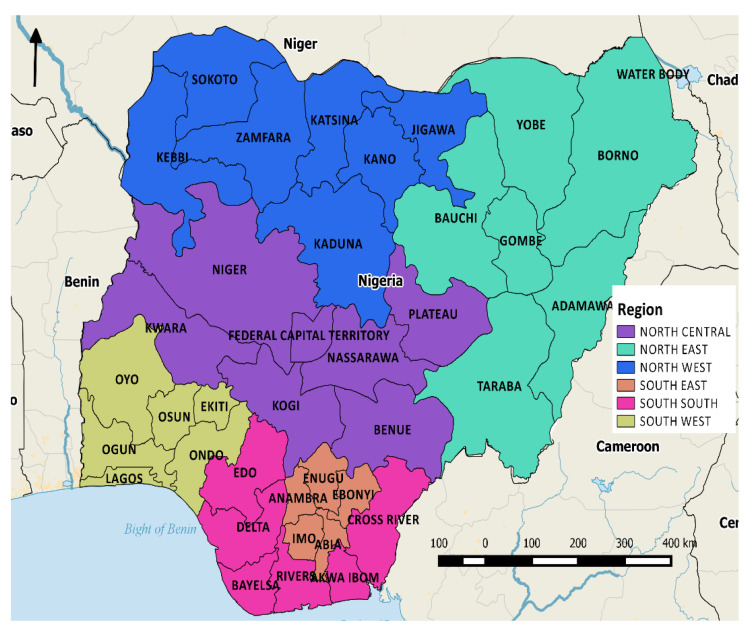
Map of Nigeria showing 36 states (plus federal capital territory) and the country’s six geopolitical regions.

Nigeria is a West African country situated between latitudes 4° and 14° N and longitudes 2° and 15° E on the Gulf of Guinea, and has a total area of 923,768 km^2^ (Figure 1). The NDHS2018 data collection occurred between 14 August and 29 December 2018, using a two-stage stratified cluster sampling framework. Nigeria’s 36 states and capital territory were separated into rural and urban areas and further grouped into six geopolitical zones, North-Central, North-East, North-West, South-East, South-West, and South-South, leading to a total of 74 sampling strata [37]. Further details of the NDHS sampling design can be found elsewhere [37]. Of the 40,666 occupied households selected for the sample, 40,427 were successfully interviewed, resulting in a response rate of 99% [37]. The resulting dataset consisted of 41,821 individual women (age 15–49) and 127,545 birth records. 

The outcome variables in this study were adverse pregnancy outcomes; birth status (stillbirth or alive, stillbirths were identified as pregnancies that did not result in the birth of a live child and included miscarriages), birth weight (children born under 2500 g were classified as LBW [1] or normal weight otherwise) and duration of pregnancy (term birth vs. preterm birth). Preterm births included those born before 37 weeks or 8.515 months of pregnancy [38]. The main exposure variables measuring HAP were cooking fuel type and mother’s smoking habit. Respondents were asked about the primary source of fuel for household cooking. Responses included crops, animal dung, charcoal, kerosene, straw/shrubs/grass, coal, wood, liquefied petroleum gas (LPG), biogas, electricity, and natural gas. We classified LPG, biogas, electricity, and natural gas as “clean fuel” and the rest as “unclean” as per WHO guidelines [7]. 

Other variables explored include socio-economic, demographic, and geographical factors such as mother’s age, educational attainment (no education, primary, secondary, higher), and wealth quintiles. As some mothers were identified with multiple birth records, we also controlled for the mother’s effect on birth outcomes. This allowed us to account for the correlation between children with the same mother. We used the states to identify the effect of residence on adverse birth outcomes and the geopolitical regions as a fixed effect. See Table 1 for the descriptive summaries of the characteristics of the study participants.

### 2.2. Statistical Analysis

We used frequencies and percentages to describe categorical variables and means and standard deviations for continuous variables. We tested the association between cooking fuel type and categorical variables (smoking status, education, wealth status) using Pearson’s chi-square test or Fisher’s exact test and presented the estimated prevalence ratios (PR) with their 95% confidence interval (CI) [39,40]. We use a *t*-test to assess the difference in mother’s age across adverse birth outcomes. 

A Bayesian approach to generalized structured additive regression (STAR) [41,42,43,44] was then used to estimate the association between adverse birth outcomes and HAP while adjusting for other predictors and region-specific terms. For a specific health outcome for child *i*, $Y_{i}$, the structured additive regression model with predictors is formulated as:

(1) $$\mathrm{Pr}\left(Y_{i}|\mathrm{predictors}\right)=h\left(\eta_{i}\right)$$

Using an appropriate link, *h*(.), such as the probit link for ease of interpretation,

(2) $$\begin{array}{c} \eta =\gamma_{0}+\gamma_{1}\mathrm{fuel}+\ldots +\gamma_{k}\mathrm{COV}+f_{1}\left({\mathrm{mother}}^{’}s\;\mathrm{cluster}\right)+f_{2}\left({\mathrm{mother}}^{’}s\;\mathrm{age}\right)\\ +f_{unstr}\left(\mathrm{STATE}\right)+f_{str}\left(\mathrm{STATE}\right) \end{array}$$

where $\gamma_{1}\ldots \gamma_{k}$ represents the fixed effect of the exposure cooking fuel variable and other covariates (COV) such as mother’s age, mother’s education, household wealth quintile, geopolitical regions) modelled with Gaussian priors for continuous variables and diffuse priors for categorical variables; function $f_{1}\;$represents a random effect term for mother’s cluster identification; $f_{2}$ is the nonlinear effect of mother’s age on health outcome modelled with Penalize splines. Bayesian Penalized-spline priors was specified for the regression coefficients, $f_{1}$ for mother’s random effect and $f_{2}$, for the nonlinear effects mother’s age with weakly informative inverse Gamma prior for the precision hyperpriors. $f_{unstr}$ is the unstructured spatial effect with random effects term for the states in Nigeria, while $f_{str}$ represents the structured spatial effect. We assumed i.i.d Gaussian random effects for the states in Nigeria in the unstructured spatial effect, $f_{unstr}\left(\mathrm{STATE}\right)$ and specified Markov random field prior for structured spatial effect, $f_{str}\left(\mathrm{STATE}\right)$.

A unique model was fitted for each adverse birth outcome: birth status, birth weight, and pregnancy duration. All outcome variables were analyzed using STAR multivariable logistic regression. Four models from simple to complex formulations were considered to select important predictors for each adverse birth outcome. Positive coefficients correspond to an increased risk for the modelled outcome. Model 1 includes the exposure variable, types of cooking fuel, and spatial components (unstructured and structured). The mother’s random effect was added to Model 1 to form Model 2. An additional nonlinear effect of the mother’s age effect was added to formulate Model 3. For the fourth model (Model 4), we included additional covariates, mother’s education, and geopolitical regions. We excluded household wealth quintiles from the multivariable Model 4, due to issues with collinearity with cooking fuel. 

The Bayesian models were fitted via Markov Chain Monte Carlo (MCMC), carrying out 25,000 iterations with a burn-in of 5000. Model selection was based on the smallest deviance information criterion (DIC); we also report the posterior mean of deviance ($\bar{D}$) and the effective number of parameters (pD). The posterior mean (p.mean) and 95% credible intervals (CrI) were presented for the parameters included in the model [45,46]. 

All statistical analyses were implemented in R software version 3.6.2 [47], with package R2BayesX [41,42] and based on complete case data. 

## 3. Results

### 3.1. Descriptive Summaries

**Figure 2:**
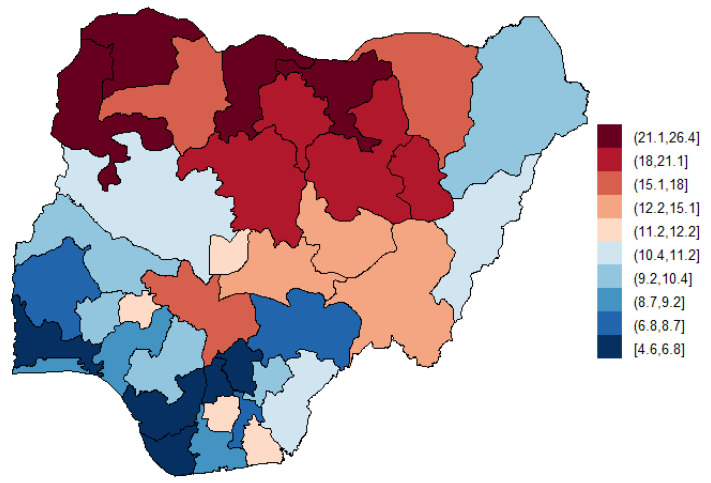
Geographical distribution of adverse birth outcomes. The prevalence is reported as the number of adverse birth outcomes per 100 pregnancies. Adverse birth outcomes include stillbirth, preterm or low birth weight (LBW).

**Figure 3:**
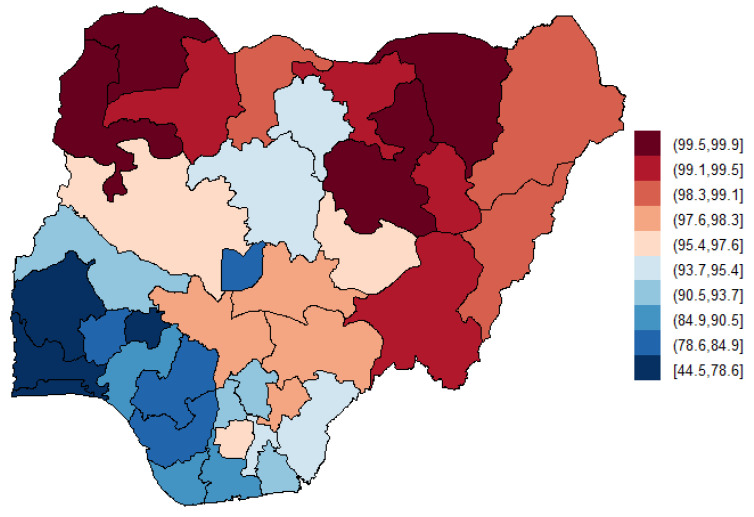
Geographical distribution of prevalence (%) use of unclean cooking fuel in Nigeria.

Table 1 presents the descriptive summaries of the study population. Of the 41,821 women age 15–49 years surveyed (mean ± SD, 35.9 ± 7.9), 89.3% used unclean cooking fuel as their primary source of cooking energy; however, only 0.2% of the respondents were smokers. A total of 127,545 birth records were included in the final analysis, out of which 14.3% (18,220) of all pregnancies resulted in stillbirth, 7.3% (562) were identified as LBW while 1.0% (408) of the children were born prematurely. There were significant associations between cooking fuel type and mother’s age, mother’s education, wealth quintiles, mother’s smoking status, pregnancy duration, birth status, and geographical regions (Table 1). For example, compared to non-smoker, the prevalence ratio (PR) of smokers in households exposed to unclean cooking fuel was 0.50 (95% CI: 0.30, 0.83) compared to households using clean fuel. Similarly, compared to alive birth status, the prevalence of stillbirth status was higher (PR = 2.40, 95% CI: 2.21, 2.62) for household exposed to unclean cooking fuel compared to those in households using clean cooking fuel. Households with low birthweight were 17% more likely to use unclean cooking fuel, (PR = 1.17, 95% CI: 0.96, 1.43) however, this result was not statistically significant. In contrast, households with premature birth were 63% less likely to use unclean fuel (PR: 0.47, 95% CI: 0.36, 0.62). Figure 2 shows the rate of adverse birth outcomes per 100 pregnancies across the Nigerian states. Figure 3 shows the percentage of households primarily using unclean cooking fuel. Northern states appeared to have higher rates of ABO and higher dependence on unclean cooking fuel.

### 3.2. Association between HAP Exposure and Adverse Birth Outcomes 

Table 1 and Table S1 summarize the relationship between the exposure variables and the pregnancy outcome variables of interest independently.

#### 3.2.1. Prevalence of Stillbirth 

Among pregnancies that resulted in stillbirth (17,598 + 503), 97.2% were from households with mothers who used unclean cooking fuel and 2.8% of the pregnancies were from households with mothers who used clean cooking fuel (Table 1 and Table S1). There was a significant association between stillbirth and cooking fuel type (*p* < 0.001). Lower wealth quintiles, older age, and low education levels were associated with a larger proportion of reported stillbirths (*p* < 0.001). On the other hand, smoking was not associated with birth outcomes (Table S1). 

#### 3.2.2. Birth Weight (Low Birth Weight vs. Normal Weight)

About 7.6% (442/5815) of mothers who used unclean fuel in this study had children born with LBW compared to 6.5% (113/1746) of births from mothers who use clean fuel. However, there was no significant association between the type of cooking fuel and birth weight. The bivariate analysis shows that only the mother’s age and education level are independently significantly related to LBW (Table S1). Mothers who have not attained a secondary or higher education level had an increased risk of an underweight birth. The smoking status of the mother was not significantly associated with any ABO.

#### 3.2.3. Duration of Pregnancy (Preterm vs. Term Birth)

Preterm births accounted for 1.0% (408/39,738) of births in this study. Surprisingly, the proportion of preterm births in households where mothers used unclean fuel (0.95%, 345/35,972) was significantly less than households where mothers used clean fuel (2.07%, 59/2856). Wealth status, education levels, and mother’s age were significantly associated with preterm outcomes (Table S1). 

### 3.3. Predictors of Adverse Birth Outcomes

Due to the small sample of mothers with smoking status, we focused only on cooking fuel as the main exposure variable. As shown in Table S2, the model diagnostic statistics for all models considered in this study for each birth outcome indicates that Model 4 provides the best fit based on smaller DIC values for birth status and birth weight except the duration of pregnancy where Model 3 had the smallest DIC value. Table 2 presents the estimated posterior means and the 95% credible intervals (CrI) for Model 4 (which includes spatial components, mother’s random effect term, the nonlinear effect of mother’s age, and other covariates such as mother’s education and geopolitical regions) for each ABO.

**Figure 4:**
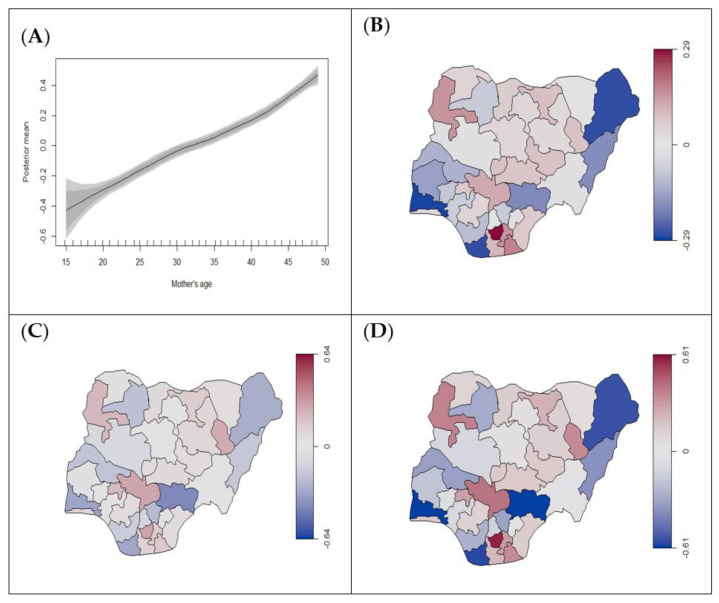
Estimated posterior means of (**A**) nonlinear effects of mother’s age (black line) on birth status (stillbirth vs. alive) with 95% and 80% credible intervals, (**B**) structured, (**C**) unstructured, (**D**) total spatial effects for the state. The scales in (**B**–**D**) represents the range of the posterior mean estimates of the spatial effect showing states with risk of stillbirth. The blue colour indicates low estimates, while the red colour signifies the states with high estimates.

**Figure 5:**
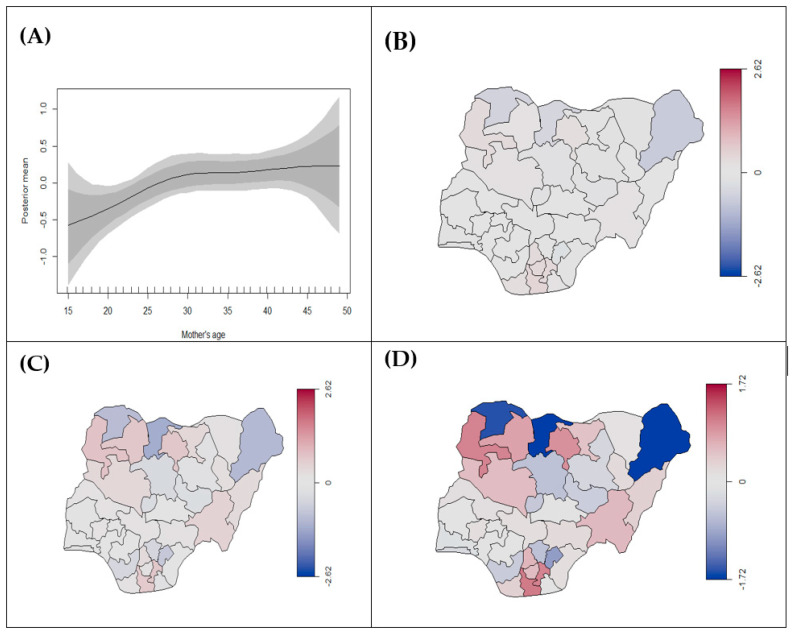
Estimated posterior means of (**A**) nonlinear effects of mother’s age (black line) on birth weight (low birth weight vs. normal) with 95% and 80% credible intervals, (**B**) structured, (**C**) unstructured, (**D**) total-spatial effects for the state. The scales in (**B**–**D**) represents the range of the posterior mean estimates of the spatial effect showing states with risk of low birthweight. The blue colour indicates low estimates, while the red colour signifies the states with high estimates.

**Figure 6:**
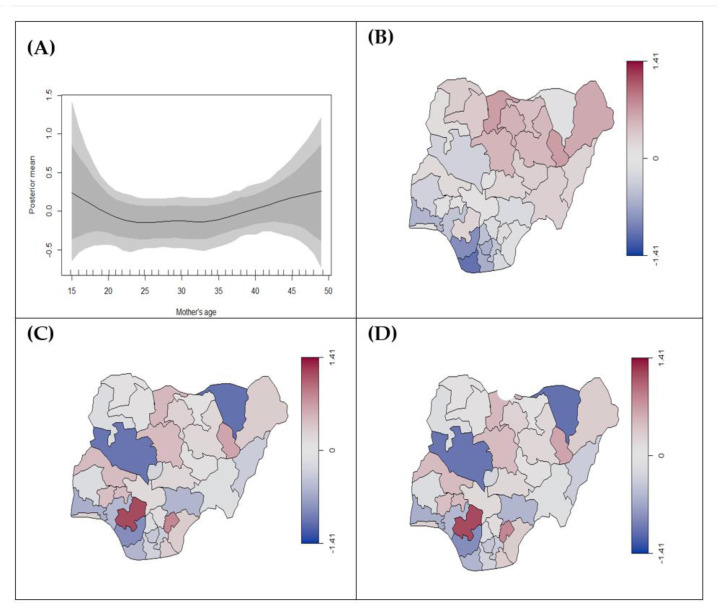
Estimated posterior means of (**A**) nonlinear effects of mother’s age (black line) on pregnancy duration (preterm vs. term) with 95% and 80% credible intervals, (**B**) structured, (**C**) unstructured, (**D**) total spatial effects for the state. The scales in (**B**–**D**) represents the range of the posterior mean estimates of the spatial effect showing states with risk of preterm birth. The blue colour indicates low estimates, while the red colour signifies the states with high estimates.

Results from Model 4 show that mothers using unclean cooking fuel are significantly at higher risk of stillbirth (0.14, 95% CrI: 0.08, 0.20) after adjusting for age, education, and region (Table 2). It also indicates that an increased level of education is protective against stillbirth, while an increase in mother’s age significantly increases the risk of stillbirth (Figure 4A). Southern states tended to have a decreased risk of stillbirth and northern states had an increased risk (Figure 4B–D). However, the use of unclean cooking fuel by mothers was not significantly associated with any low birth weight and preterm birth. Women with higher education had an increased risk of preterm compared to no education but no association was found for low birth weight. Although there was a notable increase in the risk of low birth weight and preterm birth with an increase in mother’s age, it was not statistically significant (Figure 5A and Figure 6A). Spatial disparities were noted in Figure 5B–D and Figure 6B–D, generally suggesting increased risk in the northern parts of Nigeria.

## 4. Discussion

We have shown that 89.3% of the mothers included in this study primarily used unclean fuel for cooking. This suggests that a substantial proportion of the population is still dependent on unclean cooking fuel for cooking in Nigeria. This is a significant public health concern due to the associated risks associated with unclean cooking fuel [5,6,7,8,9,10,16]. In this study, we investigated the association between HAP and adverse birth outcomes, while accounting for geographical heterogeneity and mother’s effect in our data. We were able to control for the mother’s individual effect and analyze stillbirth, LBW, and preterm outcomes. In the bivariate analysis, unclean cooking fuel was associated with a higher risk of stillbirth and protective of preterm birth. This finding supports a previous study based in Argentina that concludes that lower socio-economic status and education levels are associated with higher rates of LBW and lower rates of preterm birth [15]. 

Our result indicates that HAP was associated with an increased risk of stillbirth but not for LBW and preterm birth. Overall, northern states had a higher rate of unclean cooking fuel use and a higher rate of adverse birth outcomes. Northern states were associated with a higher proportion of stillbirths and LBW than the southern states. Previous studies conducted in Nigeria showed a link between HAP and childhood acute respiratory tract infections, which was a leading cause of deaths in children under 5 years [48,49]. Our study extends these results to include the effects of HAP in Nigeria to adverse birth outcomes. This study contributes to previous research on HAP’s association with pregnancy outcomes. Relevant literature offers contradictory findings on the significance of unclean cooking fuel when analyzing LBW. In a meta-analysis, it was concluded that indoor air pollution increased the risk of LBW [27]. A study in Bangladesh reported that indoor solid fuel use was significantly associated with LBW, but not with neonatal mortality or stillbirth when controlling for demographic variables [22]. Our study concluded that HAP did not significantly increase the risk of LBW. This may be due to the low prevalence of smoking in our sample, as smoking is correlated to increased risk for LBW [50]. This finding coincides with a Ghanaian cohort study that failed to support an association between HAP and LBW after adjusting for confounding variables [25].

Our study suggests that HAP is significantly associated with an increased risk for stillbirth and is supported by a recent meta-analysis that concluded that HAP increased the risk of stillbirth in developing countries [27]. However, other studies did not find a significant relationship between HAP and stillbirth [22,25].In a cross-sectional study using the 2007 Ghana Maternal Health Survey, the authors concluded that unclean cooking fuel could be on the causal pathway between lower socio-economic status and stillbirth [24]. It is hypothesized that smoke produced from unclean cooking fuels contains pollutants, including carbon monoxide, which can be inhaled and absorbed into the mother’s blood and possibly cause detrimental effects on the fetus [26,27]. Our study suggests that these effects on the fetus can result in an increased risk for stillbirth. 

Poverty has also been suggested to be a key factor in preventing access to clean fuel but also compounds the burden associated with HAP by worsening access to adequate health care [51]. This may be a contributing factor to the regional disparity seen between the mostly lower socio-economic demographic of the North compared to the mostly higher socio-economic demographic of the South. For example, a previous study concluded that the Northern region had the highest prevalence of underutilization of antenatal care services [52,53,54] and lowest immunization uptake [53,55,56]. When geographic variation is controlled for in the models, HAP continues to significantly increase the risk of stillbirth. Addressing the use of unclean cooking fuel in Nigeria may lead to decreased rates of stillbirth.

We acknowledge the following limitation in this study. The response rates for some adverse birth outcomes were relatively low. There was a 100% response rate for stillbirth outcomes, but there were low response rates for birth weight (6.1%) and pregnancy duration (31.2%). The smoking status of the mother was only recorded in a small proportion (0.2%) of mothers who identified as smokers. Reporting pregnancy in duration in months could introduce measurement bias in our analysis. Measuring pregnancy duration in weeks would result in a more accurate and sensitive model to variability in pregnancy duration. We also could not distinguish when the pregnancy ended. There may be different associations for perinatal mortality and miscarriages if analyzed separately. Amount of time spent around HAP could contribute to the magnitude of its effect on adverse birth outcomes, but we were unable to include this variable in this study. We could not control for all possible confounding variables such as access to medical services, medical history, and BMI. 

Despite these limitations, this study has several strengths. As the study was based on a large representative dataset, the 2018 Nigeria DHS, we are confident that our sample is an excellent representation of the Nigerian population. The use of STAR models for analysis allows for flexible modelling of possible nonlinear effects of independent variables and the geographical effects of the data [41,42,43,57].

## 5. Conclusions

This paper studied the association between HAP and adverse birth outcomes using the 2018 NDHS. It highlighted the risk of adverse birth outcomes due to mothers using unclean cooking fuel. In order to decrease the prevalence of adverse birth outcomes in Nigeria, efforts should address the dependence on unclean cooking fuel. Disparities in Nigerian states account for disproportionate risks of stillbirth and LBW, even when the effects of wealth and education are controlled for. This shows that decreasing national levels of adverse birth outcomes depends on working toward addressing the disparities between states.

Further research should be performed to analyze the effects that our study could not control for, such as access to prenatal medical services, and the mother’s medical history. This includes accounting for a combination of different fuels instead of studying only the primary cooking fuel. Analyzing dose-response using the combination of cooking fuels would clarify the strength of the relationship between HAP and adverse birth outcomes. As our study is a cross-sectional study, we cannot analyze causal pathways between HAP, adverse birth outcomes, and other explanatory variables. Further research should be done to study these causal pathways and include a larger selection of adverse birth outcomes, as we limited our study to three outcomes. 

## Figures and Tables

**Table 1:** Descriptive summaries of the study participants.

Characteristics	*n* (%)			PR (95% CI) ^b^	*p*-Value ^b^
	Overall	Clean Cooking Fuel ^a^	Unclean Cooking Fuel ^a^		
		4410 (10.69%)	36,846 (89.31%)		
Demographic Variable (Mothers or Household) ^c^	41,821				
Mother’s Age, mean ± SD	35.9 ± 7.9	29.82 ± 9.04	29.1± 9.8	NA	<0.0001
Mother’s education					
No education	14,398 (34.43)	115 (0.28)	14,173 (34.35)	ref	<0.0001
Primary education	6383 (15.26)	252 (0.61)	6062 (14.69)	0.20 (0.16, 0.24)	
Secondary education	16,698 (39.93)	2189 (5.31)	14,219 (34.47)	0.05 (0.04, 0.06)	
Higher education	4342 (10.38)	1854 (4.49)	2392 (5.80)	0.01 (0.01, 0.01)	
Household wealth quintiles ^c^					
Poorest	7747 (18.52)	5 (0.01)	7682 (18.62)	ref	<0.0001
Poorer	8346 (19.96)	11 (0.03)	8243 (19.98)	0.49 (0.15, 1.34)	
Middle	8859 (21.18)	91 (0.22)	8934 (20.93)	0.06 (0.02, 0.14)	
Richer	8840 (21.13)	656 (1.59)	8020 (19.44)	0.01 (0.00, 0.02)	
Richest	8029 (19.20)	3647 (8.84)	4267 (10.34)	0.00 (0.00, 0.00)	
Mother’s exposure variable ^c^					
Smoking Status					
Smoker	96 (0.23)	18 (0.04)	75 (0.18)	0.50 (0.30, 0.83)	0.0111
Non-smoker	41,725 (99.77)	4392 (10.65)	36,771 (89.13)	ref	
Adverse birth outcome ^d^	127,545				
Birth status					
Alive	109,325 (85.701)	7626 (6.03)	100,669 (79.65)	ref	<0.0001
Stillbirth	18,220 (14.28)	503 (0.40)	17,598 (13.92)	2.40 (2.21, 2.62)	
Birth weight ^e^					
Normal weight (≥2500 g)	7166 (92.73)	1633 (21.60)	5373 (71.05)	ref	0.1250
Low birth weight (<2500 g)	562 (7.27)	113 (1.49)	442 (5.85)	1.17 (0.96, 1.43)	
Duration of pregnancy ^f^					
Term birth	39,330 (99.00)	2856 (7.28)	35,972 (91.69)	ref	<0.0001
Premature	408 (1.00)	59 (0.15)	345 (0.88)	0.47 (0.36, 0.62)	
Geographical variable ^c^					
Region					
North-Central	7772 (18.60)	607 (1.47)	7074 (17.15)	7.21 (6.54, 7.97)	<0.0001
North-East	7639 (18.30)	86 (0.21)	7453 (18.07)	53.65 (43.37, 67.29)	
North-West	10,129 (24.20)	376 (0.91)	9703 (23.52)	15.98 (14.24, 17.97)	
South-East	5571 (13.30)	390 (0.95)	5004 (12.13)	ref	
South-South	5080 (12.10)	827 (2.00)	4181 (10.13)	3.13 (2.86, 3.43)	
South-West	5630 (13.50)	2124 (5.15)	3431 (8.37)	7.94 (7.08, 8.93)	

^a^ Missing 565 cases. ^b^ Prevalence ratios (PR), comparing unclean vs. clean cooking fuel; *p*-value based on chi-square test. ^c^ At mother or household level, *n* = 41,821. ^d^ At child level, *n* = 127,545. ^e^ Missing birth weight records on 119,817 (93.9%) births. ^f^ Missing duration of pregnancy record on 87,807 (68.8%) pregnancies. Ref: Reference category.

**Table 2:** Parameter posterior mean and 95% credible interval (CrI) from multivariable analysis (Model 4) of adverse birth outcomes.

Characteristics	Birth Status: Stillbirth vs. Alive	Birth Weight: Low vs. Normal	Pregnancy Duration: Preterm vs. Term
	*p* Mean (95% CrI)	*p* Mean (95% CrI)	*p* Mean (95% CrI)
Cooking Fuel			
Clean (ref)			
Unclean	0.14 (0.08, 0.20)	−0.09 (−0.31, 0.10)	−0.01 (−0.33, 0.31)
Education			
No education (ref)			
Primary	−0.07 (−0.10, −0.03)	0.22 (−0.09, 0.49)	−0.04 (−0.33, 0.34)
Secondary	−0.23 (−0.26, −0.19)	0.37 (0.08, 0.68)	0.14 (−0.13, 0.42)
Higher	−0.39 (−0.45, −0.32)	0.36 (0.06, 0.65)	0.58 (0.24, 1.94)
Region			
North-Central (ref)			
North-East	0.262 (0.01, 0.44)	0.10 (−0.622, 0.88)	−0.01 (−0.64, 0.75)
North-West	0.36 (−0.02, 0.60)	−0.52 (−1.21, 0.19)	−0.42 −0.99, 0.09)
South-East	−0.16 (−0.44, 0.07)	0.23 (−0.57, 0.94)	−0.06 (−0.86, 0.70)
South-South	−0.15 (−0.35, 0.05)	0.20 (−0.44, 0.77)	−0.11 (−1.01, 0.57)
South-West	−0.10 (−0.34, 0.15)	−0.02 (−0.73, 0.67)	0.15 (−0.74, 0.77)

## Data Availability

The survey data sets used in this paper is publicly available from the DHS Program (www.dhsprogram.com).

## Supplementary Materials

The following are available online at https://www.mdpi.com/1660-4601/18/2/634/s1, Table S1: Bivariate analysis of adverse birth outcomes and HAP exposure, mother’s level, and household characteristics. Table S2: Criterion for model selection.
