# Supplementary material for: Adverse Birth Outcomes Due to Exposure to Household Air Pollution from Unclean Cooking Fuel among Women of Reproductive Age in Nigeria
Source: Int J Environ Res Public Health. 2021 Jan 13;18(2):634. doi: 10.3390/ijerph18020634 (PMC7828613; doi:10.3390/ijerph18020634)
Supplement: Supplementary file 1 [file ijerph-18-00634-s001.pdf]

Supplementary

Table S1. Bivariate analysis of adverse birth outcomes and HAP exposure, mother's level, and household characteristics.

Characteristics	Birth Status			Birth Weight			Pregnancy Duration		
	**Stillbirth, *n* (%)**	**Alive, *n* (%)**	**PR (95% CI)**	**LBW, *n* (%)**	**Normal, *n* (%)**	**PR (95% CI)**	**Preterm, *n* (%)**	**Term, *n* (%)**	**PR (95% CI)**
Mother's Age, mean ± SD	37.0 ± 7.9	35.7 ± 7.93	<0.0001	29.9 ± 6.3	30.6 ± 6.1	0.012	30.8 ± 6.9	29.9 ± 6.9	0.011
Smoking Status									
Non-Smoker	18,187 (99.8)	109107 (99.8)	0.671	559 (99.5)	7147 (99.7)	0.212	408 (100)	39,246 (99.8)	1
Smoker	33 (0.2)	218 (0.2)		3 (0.5)	19 (0.3)		0 (0)	84 (0.2)	
Cooking Fuel									
Clean	503 (2.8)	7626 (7)	<0.0001	113 (23.4)	1633 (23.3)	0.125	59 (14.6)	2856 (7.4)	<0.0001
Unclean	17,598 (97.2)	100,669 (93)		442 (76.6)	5373 (76.7)		345 (85.4)	35,972 (92.6)	
Wealth Quintile									
Poorest	6072 (33.3)	25,076 (22.9)	<0.0001	27 (3.8)	226 (3.2)	0.107	73 (17.9)	9403 (23.9)	<0.0001
Poorer	5206 (28.6)	24,242 (22.2)		46 (620.2)	574 (620.8)		69 (16.9)	9003 (22.9)	
Middle	3545 (19.5)	23,575 (21.6)		115 (19.5)	1367 (19.1)		84 (20.6)	8337 (21.2)	
Richer	2252 (12.4)	20,958 (19.2)		171 (29.4)	2097 (29.3)		90 (22.1)	7130 (18.1)	
Richest	1145 (6.3)	15,474 (14.2)		203 (40.1)	2902 (40.5)		92 (22.5)	5457 (13.9)	
Education									
No education	11,806 (64.8)	51,893 (47.5)	<0.0001	87 (8.5)	560 (8.8)	< 0.0001	135 (33.1)	18,071 (45.9)	<0.0001
Primary	3318 (18.2)	21,993 (20.1)		83 (12.8)	898 (12.5)		59 (14.5)	6180 (15.7)	
Secondary	2599 (14.3)	28,157 (25.8)		269 (54.9)	3920 (54.7)		145 (35.5)	12,116 (30.8)	
Higher	497 (2.7)	7282 (6.7)		123 (24.9)	1788 (24.25)		69 (16.9)	2963 (7.5)	
Region									
North Central	2413 (13.2)	19,243 (17.6)	<0.0001	124 (22.1)	1480 (20.7)	< 0.0001	78 (19.1)	6773 (17.2)	<0.0001
North East	4114 (22.6)	22,179 (20.3)		49 (8.7)	605 (7.1)		86 (21.1)	8268 (21.0)	
North West	8510 (46.7)	31,418 (28.7)		109 (19.4)	528 (7.4)		69 (16.9)	12,102 (30.8)	
South East	1166 (5.6)	12,906 (11.6)		85 (15.1)	1900 (26.5)		49 (12.0)	4455 (11.3)	
South South	1011 (5.5)	11,425 (10.5)		63 (11.2)	1091 (15.2)		36 (8.8)	3687 (9.4)	
South West	1006 (5.5)	12,154 (11.1)		132 (23.5)	1662 (23.2)		90 (22.1)	4045 (10.3)	

Table S2. Criterion for model selection.

Model	Description	Model Diagnostic	Birth Status	Birth Weight	Pregnancy Duration
Model 1	Includes types of cooking fuel+spatial component	Deviance ($\bar{D}$)	99,681.89	3710.40	4300.44
		(pD)	36.62	31.75	28.54
		DIC	99,755.14	3773.90	4357.53
Model 2	Model 1 + random effect	Deviance ($\bar{D}$)	81,626.45	1562.13	1123.85
		(pD)	7045.53	859.53	673.25
		DIC	95,717.51	3281.19	2470.36
Model 3	Model 2 + nonlinear effect of age	Deviance ($\bar{D}$)	81,898.82	1495.81	1129.00
		(pD)	6792.76	873.25	676.42
		DIC	95,484.45	3242.32	2481.84
Model 4	Model 3 + education and geopolitical regions	Deviance ($\bar{D}$)	81,931.41	1423.36	1147.37
		(pD)	6686.12	885.32	680.50
		DIC	95,303.67	3194.00	2508.36
